# Pharmacy Students’ Perceptions of Self-Reflection and Peer and Educator Feedback on the Development of Patient Counselling Skills: A Qualitative Analysis

**DOI:** 10.3390/pharmacy14020041

**Published:** 2026-03-03

**Authors:** Jessica Pace, Andrew Bartlett, Tiffany Iu, Jonathan Penm

**Affiliations:** 1 Sydney Pharmacy School, Faculty of Medicine and Health, The University of Sydney, Sydney, NSW 2006, Australia; andrew.bartlett@sydney.edu.au (A.B.); tiiu2693@uni.sydney.edu.au (T.I.); jonathan.penm@sydney.edu.au (J.P.); 2 Department of Pharmacy, Prince of Wales Hospital, Sydney, NSW 2031, Australia

**Keywords:** patient counselling, feedback, self-reflection, peer assessments, self-assessment

## Abstract

(1) Background: Simulation is an effective way to develop practical pharmacy skills; combining simulation and self-reflection can increase impacts on learning. While existing literature highlights the benefits of reflection in developing self-awareness, critical thinking, and professional skills, there are few specific insights into how reflective practices enhance learning in patient counselling role-plays. This study aimed to explore pharmacy students’ perceptions of self-reflection and peer and educator feedback on the development of patient counselling skills. (2) Methods: Thematic analysis of student reflections on learning in patient counselling activities. Responses to four structured self-reflection prompts were collected and analyzed thematically. (3) Results: Reflections from 201 students were analyzed. We identified four themes and ten associated subthemes: impact of peer feedback (subthemes supportive peer dynamics and developing a personal counselling style through peer practice); impact of self-reflection and assessment (subthemes goal setting through self-reflection and video review as a tool for skill refinement); impact of educator feedback (subthemes feedback variation in learning growth and addressing self-doubt); and professional identity (subthemes value pharmacists can bring, struggles in real-life practice, incorporating feedback to working opportunities, and reinforcing skills to self-reflect in future practice). (4) Conclusions: Integrating consistent, high-quality feedback from educators and peers with self-reflection in patient counselling activities is perceived as valuable to enhancing enhances students’ learning experiences and preparing them for professional practice.

## 1. Introduction

Simulation-based learning plays a crucial role in pharmacy education, offering students opportunities to hone essential skills such as patient assessment, delivering health interventions, and building mutual trust with patients [1]. Research indicates that this approach significantly enhances both verbal and non-verbal communication skills, boosts confidence, and fosters the perception of delivering high-quality patient care [2]. The integration of patient simulation into the standard curriculum has also been shown to cultivate empathy and patient-centredness, as students practice addressing patient needs and providing tailored health advice while also cultivating pharmacotherapy knowledge [3]. Experiential learning is fostered in patient simulation practice, immersing students in realistic scenarios that allow them to apply theoretical knowledge, develop clinical skills, and adapt to the dynamic needs of patient care. Additionally, the structured yet challenging nature of simulation creates a rich learning environment, as it requires higher level thinking for clinical reasoning and information gathering [4]. These activities offer a controlled setting for individualised competence assessments, enabling students to refine their communication strategies and medication counselling techniques through both peer and educator feedback. This provides students room to develop key skills for future practice and assists them in identifying their strengths and weaknesses. Overall, simulation-based education is a well-established method for equipping pharmacy students with the practical skills necessary for future professional practice.

To maximise the learning opportunities in simulation-based activities, encouraging students to reflect on their performances is essential for building skills and fostering personal growth [5]. Reflection enhances pharmacy students’ critical thinking and introspective learning and provides deeper insights into their strengths and weaknesses as they progress in their educational journey. Consistent and thoughtful reflection empowers self-directed learning, ultimately helping students develop the cognitive skills needed to become competent, self-aware, and adaptable practitioners. With a mindset of continuous self-improvement, pharmacy students can approach change with openness and view challenges as growth opportunities, preparing them to adapt to the ever-evolving scope of professional pharmacy practice. The benefits of reflection in pharmacy education are well-documented, leading to the integration of reflective tools into curricula [6]. Common methods, such as diaries, blogs, reflective statements, and video or audio recordings, help students externalise thoughts, overcome barriers, and self-evaluate their counselling performance [7]. These practices encourage pharmacy students to analyse experiences, decisions, and actions on a deeper level, underscoring the importance of reflection in simulation-based tasks.

When accompanied with reflection, the learning outcomes from simulation-based tasks are enhanced in students in multiple ways. For example, structured reflective practices help develop critical skills needed to make more appropriate therapeutic decisions, ultimately improving future patient outcomes, and raise awareness of factors influencing clinical decisions, such as patient values, motivation, and psychosocial issues, which may present as potential challenges in real-life practice [8]. By recognising these patient factors through reflecting on counselling, students can effectively practice the skill of tailoring health interventions and treatments to individual circumstances. Moreover, students can also recognise potential cognitive biases and personal attitudes that may have influenced their decisions through reflecting on their own cognitive processes. A study on nurse practitioners’ reflection during simulated patient encounters revealed that reflection encouraged their desire to learn more about diverse patient needs, facilitated awareness and potentially mitigated biases in clinical practice [9]. Further, reflection not only provides students with opportunities to enhance core communication skills and build confidence for more effective patient counselling but also encourages self-directed learning and the development of autonomy [9]. Reflection allows students to learn intrinsically during simulated interactions and identify their unique skill sets, ultimately allowing students to envision their future roles with greater confidence. Creating a reflective loop after simulation-based tasks allows pharmacy students to practice self-awareness and personal improvement, in hopes that this practice can be brought to future professional development activities. Overall, while existing literature highlights the benefits of reflection in developing self-awareness, critical thinking, and professional skills [5], it often lacks specific insights into how reflective practices enhance learning in the context of patient counselling role play activities. Therefore, the aim of this study was to explore pharmacy students’ perceptions on self-reflection and peer and educator feedback on the development of patient counselling skills.

## 2. Materials and Methods

### 2.1. Study Setting

PHAR3825 Pharmaceutical Skills and Dispensing B is a mandatory third-year unit in the University of Sydney’s four-year Bachelor of Pharmacy program, the undergraduate pathway to professional registration. The unit is designed to extend students’ therapeutic knowledge by developing their capability to counsel patients on the safe and effective use of prescribed medicines. Prior to this unit, students have practised simulated counselling with peers on over-the-counter (OTC) products during their first and second years.

Teaching and learning activities revolve around simulated counselling role-play activities, where students conduct and observe simulated counselling sessions with peers and demonstrators, combined with video recording and review. Across the semester, each student completes seven individual counselling scenarios with a tutor (also referred to as a demonstrator) and observes 28 additional peer-led scenarios, from which the seven assessed cases are selected. In every class session, students record themselves counselling a peer on a designated case and upload the video to the unit’s Canvas learning management system (LMS).

Case scenarios for the unit are selected from the 50 most frequently dispensed medicines on Australia’s Pharmaceutical Benefits Scheme (PBS) [10]. Students are guided to follow a consistent approach to counselling, built around the “three prime questions”:

1. What did the doctor say this medicine is for?
2. How did the doctor say to take it?
3. What did the doctor say to expect [11]?

In earlier offerings of the unit, students were permitted to choose their own peers for both participation and feedback, and feedback from peers was released without prior review. Following insights from a previous course evaluation, a new system was introduced in 2022: videos uploaded to the LMS are now randomly allocated to another student for marking using a standard rubric, with all peer feedback anonymised and reviewed by teaching staff before release.

Students also undertake a self-assessment of each counselling performance, using the same rubric applied to peer reviews, and reflect on their progress to identify personal learning needs. After each simulated session, they respond to a set of structured reflection questions, and at the conclusion of the unit they complete a 500-word reflective statement. The reflection questions used throughout the semester and in the final assessment are presented in Table 1.

**Figure 1:**
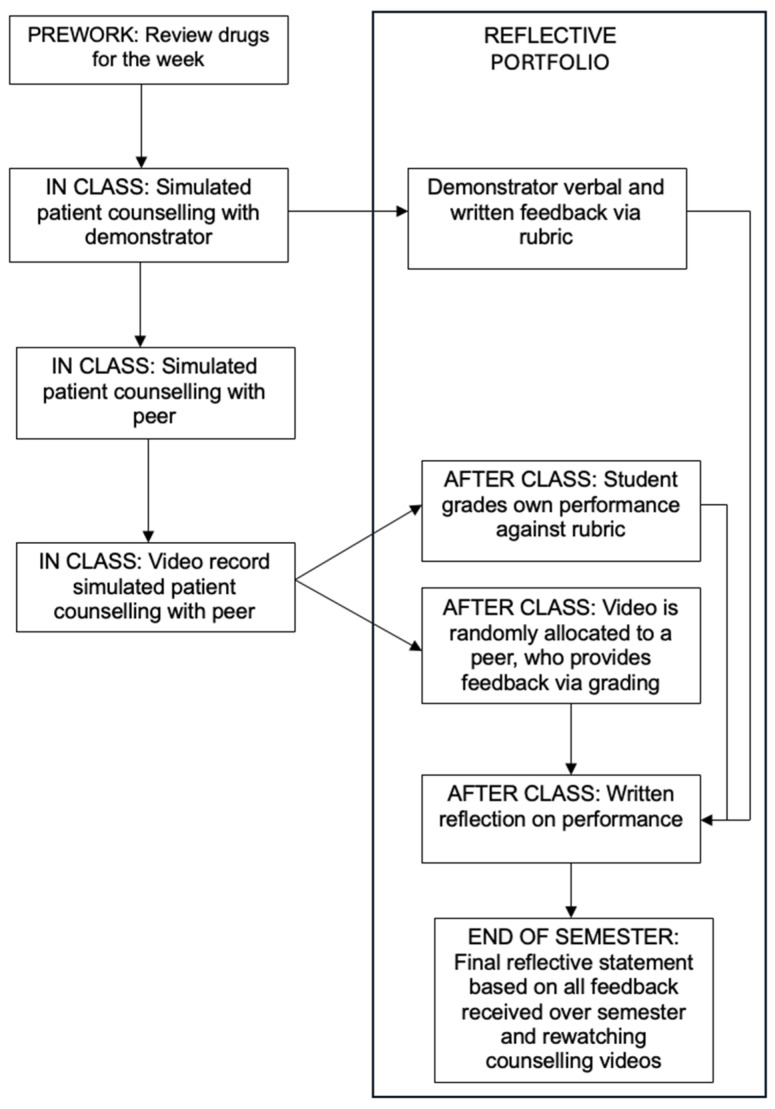
Structure of the learning activity.

Figure 1 provides an overview of the structure of the learning activity. The learning outcomes primarily include effective counselling and education of patients about medicines and disease states. The unit uses pass–fail grading (i.e., students receive a grade of satisfied requirements (SR) or failed requirements (FR) but no numerical grade at the end of the unit). All assessment tasks (e.g., counselling assessments and reflections) need to be completed satisfactorily but have a 0% weight.

### 2.2. Data Collection and Analysis

This study involved qualitative analysis of students’ final reflective statements submitted at the end of the unit for the 2022 cohort. An opt-out consent process was used, whereby students were provided with information about the study via announcements including a participant information statement on the relevant Canvas sites and given two weeks after the last announcement to notify the researchers that they did not want their reflective statements to be used in this analysis. Reflective statements for any students who had not notified the researchers were included in the analysis. These were downloaded from Canvas and deidentified before being entered into QSR International Nvivo software version 12 for data management and analysis. Each participant was assigned a number for anonymity.

Data were analysed using an iterative, inductive approach to thematic analysis, following the procedures described by Braun and Clarke [12]. This analytic method allows researchers to identify, explore, and report recurring patterns within qualitative data and to develop meaningful interpretations based on those patterns. It involves six steps: familiarisation with data, generating initial codes, searching for themes, reviewing themes, defining and naming themes, and producing the final report. All members of the research team familiarised themselves with data by reading reflective statements before initial data coding and analysis were conducted by TI. Analytic memos were created for each reflective statement, and these ideas informed the development of the coding framework. A mixture of *a posteriori* descriptive (using short words or phrases to summarise the topic of a piece of qualitative data), initial (breaking down qualitative data into discrete parts, closely examining them, and comparing them for similarities and differences), and process (using gerunds or “-ing” words to connote action in the data) coding was used [13]. This resulted in 55 codes. Related codes were then collapsed into preliminary themes. Ongoing discussions between JP, AB, JLP and TI were used to refine the coding framework and to shape, define, and label the emerging themes. Notes were maintained to document all decisions made during these meetings. Analysis proceeded until every reflective statement had been examined and thematic saturation was achieved—that is, no additional themes were identified, and existing themes were fully developed and clearly articulated [14]. TI and JLP prepared the final report, which was subsequently reviewed and endorsed by all authors. Participants did not review or comment on the findings.

Steps to support trustworthiness include documentation of analytic decisions through memos and meeting notes, ongoing team discussions to develop, refine, and name themes and a robust process for developing and refining the coding framework as outlined above.

The Consolidated Criteria for Reporting Qualitative Research (COREQ) checklist [15] was used to report this study (see Supplementary Materials for completed checklist).

#### Reflexivity Statement

TI was a final year female pharmacy student who had completed PHAR3825 in 2024 and had no prior relationship with participants. She received training in qualitative methods and thematic analysis before completion of this project. She was supervised in this research project by JP, JLP and AB, who are experienced pharmacy educators and pharmacists. JP and AB developed PHAR3825, and JLP and AB have since refined the teaching and learning activities and assessment structure. JP, AB and JLP have also all convened and taught and assessed students in the various iterations of the unit. Their long-standing involvement with the unit may have influenced both the data and interpretation. These influences were considered during the data analysis through team discussions and documentation of decisions (e.g., through memos and meeting notes as outlined above).

## 3. Results

A total of 203 students completed PHAR3825 in 2022. One student did not give permission for their reflective statement to be analysed, and one did not complete the assessment, leaving a total of 201 reflective statements (99% of the cohort) for analysis. A total of 131 students (=65%) were female and the median age of students was 20 years.

We identified four themes and ten associated subthemes. These are outlined in Table 2 and described in more detail below.

### 3.1. Impact of Peer Feedback on Development of Skills and Confidence

#### 3.1.1. Supportive Peer Dynamics

Peers played a crucial role in fostering a supportive learning environment, which helped students overcome nervousness and self-doubt. Having a peer who provided encouragement and feedback in a non-judgemental way helped ease performance anxiety for counselling assessments, making students more comfortable experimenting with different counselling approaches. Peer feedback was particularly effective in reducing nervousness before recorded and assessed counselling sessions as students gained confidence from peer support. The presence of supportive peers transformed what could have been a stressful experience into a collaborative learning process.

*“Practicing with peers is extremely helpful, as stakes are lower than when practicing with tutors and thus not as stressful… In my experience my partner and I were keen to help each other improve and were non-judgemental when we made mistakes.”*

Some students noted that while peers provided reassurance at times of self-doubt, their feedback was often hindered by bias or lack of critical depth. Practicing with peers and receiving feedback provides students with more ease; however, this comfort sometimes led to overly positive or less critical feedback, which limited opportunities for meaningful improvement. Students pointed out that peer feedback can sometimes lead to a false sense of security, meaning that they rely on demonstrators’ feedback more heavily for constructive criticism.

*“Throughout the semester, I found the tutor’s recommendations at the end of my counselling sessions to be more honest than my peers. Whilst my peers tended to say that I was doing great, the tutors gave me the more direct advice.”*

Students also appreciated the anonymous online peer feedback, as it provided a safe space for constructive criticism and self-improvement. Although anonymity encouraged more honest and unbiased critiques, some students flagged that pieces of feedback were not received and that the quality of feedback wasn’t assured.

*“To be completely honest, besides the feedback that I received on my last video, the feedback that I would receive from the anonymous users were not really beneficial.”*

#### 3.1.2. Developing a Personal Counselling Style Through Peer Practice

Not only did students benefit in their learning through feedback exchange with peers, but they also appreciated the unique element of observing others’ performance and learning from their communication styles. Through other’s performance, students recognise their own areas of improvement, making their learning experience more personalised and practical. Moreover, practicing with peers allowed students to experiment with different techniques, as well as analyse different communication styles and adopt new strategies.

*“For me, practicing with peers is the most effective form of learning as it incorporates feedback and actual practice. I get to acquire tips and insights into how my peers approach their counselling, which is highly beneficial to my learning. I tend to learn better with others, so this form of learning is best suited for me.”*

At the start of the semester, many students felt that they had to follow a strict, structured approach to counselling, which sometimes made their speech sound scripted or unnatural. Through practicing with peers and receiving personal feedback, students began to develop their own counselling styles. Some students preferred a conversational and empathetic approach, while others found that being direct and concise worked best. Students observed their peers’ counselling styles to tailor their own approach in a way that felt more natural and suited their personal communication style. By integrating elements they found effective, students refined their techniques to enhance patient engagement and confidence in their delivery.

*“I believe it is very important to practice with your peers. This is because you are able to learn how they counsel and implement it into your own style.”*

### 3.2. Impact of Self-Reflection and Assessment on Development of Skills and Confidence

#### 3.2.1. Goal Setting Through Self-Reflection

Self-reflection allowed students to critically assess all aspects of their counselling performance. By using the framework on the online portal, they found it effective for tracking progress and ensuring they met their previous goals. Regular self-reflection not only enhanced their motivation for continuous improvement but also strengthened their confidence as they observed tangible skill development over time.

*“Self-reflection was also helpful for my learning as sometimes I can get very caught up with my classes, lectures and don’t have time to self-reflect… By writing it down and coming back, it allows me to see if I have overcome my problems and if I should do something different next time. Also seeing if the goals I made were achievable and how I achieved it and maybe next time I could use a similar approach in solving other problems.”*

While appreciating the improvement they had as they progressed, some students thought the exercise of self-reflection became repetitive, particularly as they became more confident in their skills. As students looked for skills to refine and areas of improvement to set goals, they thought that as they progressed the opportunities for meaningful improvement became less apparent through self-reflection. Students noted that at a certain point as self-reflection continues, their counselling had improved to an extent where self-assessment provided little new insight, as they were already self-aware of their strengths and weaknesses. As a result, they continued the necessity of writing self-reflections after reviewing their performance, suggesting that the exercise was more beneficial in the earlier stages of skill development. In a sense, students have found self-reflection unnecessary to a certain point because they had internalised the skills needed to progress through self-reflection.

*“Although initially it was fantastic, beyond [the third class] it became fairly repetitive and slightly unnecessary as we had already worked on most, if not all, of our weaknesses, and our counselling was essentially “perfect” where we didn’t really need to watch it back because we would be awarding ourselves full marks.”*

#### 3.2.2. Video Review as a Tool for Skill Refinement

Many students initially found watching their own counselling videos uncomfortable, as it made them more self-conscious about their speech, body language and nervous habits. However, over time, they came to appreciate video review as a powerful self-directed learning tool. They found that seeing their own performance objectively helped them identify overlooked mistakes, adjust counselling tone, and experiment with different strategies to see what worked best. The ability to critique their own work allowed students to take ownership of their learning over time.

*“Through watching my performance in the counselling videos, I was able to evaluate my performance objectively and hence identify areas of improvement. Watching videos of myself from the first counselling session through to the last, I realised there were subtle improvements progressively which felt rewarding.”*

However, not all students found video review to be an effective learning strategy. Some felt that real-time feedback from peers and demonstrators was more immediate and actionable than reviewing recordings. Others admitted they avoided watching their videos altogether, either due to discomfort or because they felt the effort required to record, upload and review the videos outweighed the benefits. Many students also pointed out that self-reflection and sharing feedback with peers happened naturally during or immediately after the counselling sessions, making the additional step of formally reviewing and writing reflections on video recordings feel redundant. Additionally, some students struggled with the format of video review, expressing that recording sessions felt unnatural and disrupted their ability to fully immerse themselves in the counselling process. The presence of a camera made them more self-conscious, and students found themselves focusing more on performing for the recording rather than engaging authentically with the counselling task.

*“I do not think the self-reflection was beneficial our peers critique us naturally after the video counselling and most students self-reflect almost immediately after the video counselling naturally. Hence, it seems to be more of an extra step to write down what we’ve already thought of and discussed onto the portal for self-reflection.”*

### 3.3. Impact of Demonstrator Feedback on Development of Skills and Confidence

#### 3.3.1. Feedback Variation in Learning Growth

The provision of constructive and specific feedback by demonstrators helped students feel more competent in their counselling skills. Students acknowledged that not only did they point out areas of improvement, but they also reinforced what students were doing well, which reduced anxiety and enhanced self-assurance. Students trusted demonstrators’ judgement and feedback due to their expertise and professional background, allowing them to trust their progress and feel more prepared for upcoming oral assessments and real-life practice.

*“I often think that I pause for too long or say too many “ums” however when I brought up my concerns with the demonstrator, they assured me that it was not to the extent that I had thought. This allowed my confidence to improve which eventually led me to have a better flow during my counselling sessions. The demonstrator also reassured me whenever I had doubts on my performance and they eased my anxiety that I had.”*

Demonstrators also played a crucial role in providing individualised feedback to students to improve techniques on patient engagement and assessing their needs. Through strategies such as breaking information into digestible bites, and summarising key points, students practiced ensuring that patients were not just passive recipients of information but active participants in the counselling process. Many students acknowledged how easy counselling can become a one-way information dump and realised the importance of active patient engagement rather than just delivering information.

*“One way I improved in the second aspect was by taking on the advice from my marker, where I was told to “chunk and check”. This saw me improve in ensuring the patient understood the information I was giving them whilst also involving them in their own healthcare.”*

While many students acknowledged that demonstrators provided insightful and balanced feedback on both strengths and weaknesses, some students felt that the feedback could have been more critical to push them further. For these students, the encouraging tone of the feedback, while helpful for confidence building, lacked the rigor needed for deeper self-improvement. Students suggested that rotating demonstrators and incorporating more varied feedback or feedback styles might enhance learning in a broader perspective.

*“Feedback from demonstrators was useful. My demonstrator was much more encouraging than others and I believe they could have been harsher.”*

#### 3.3.2. Addressing Self-Doubt in Counselling Certain Medications

Initially, many students felt uncertain when counselling on complex or sensitive medications, particularly those related to mental health or high-risk treatments. This self-doubt stemmed from fears of giving incorrect information, struggling with patients’ questions, or lacking fluency in their explanations. However, through demonstrator feedback, students learn how to simplify complex information, use patient-friendly language, and address difficult questions with more assurance. Through their level of expertise, demonstrators provide students with clear guidance on how to approach these challenges, offering step-by-step explanations or using metaphors to break down complex topics into manageable parts. Over time, students became more comfortable addressing patient concerns and explaining use of medications, ultimately enhancing their confidence in real-world pharmacy practice.

*“There were definitely certain medications that I found uncomfortable to counsel such as anti-depressants. It always felt awkward prying into people’s lives when I didn’t know how comfortable they were with sharing that information…but after advice from the tutors I realised it’s something you just have to approach with a more casual take.”*

### 3.4. Professional Identity

#### 3.4.1. Learning About the Value Pharmacists Can Bring

Through reflections, students gained a deeper understanding of what it means to be a pharmacist, moving beyond the technical aspects of medication dispensing to recognising their broader role in patient care. Many initially viewed their responsibilities as primarily providing drug information, but as they engaged in counselling simulations, they realised the significance of their role in patient outcomes, medication safety, and healthcare accessibility.

*“PHAR3825 made me aware that pharmacy isn’t just about dispensing medication. It’s about ensuring that we are able to add value to a patient’s pharmacy experience. Value can come in many different forms like sympathy, educating them on misconceptions, creating a safe and honest space. As pharmacists we have the ability to break down any health care stigmas and give our patients the care and education they deserve.”*

A key realisation was that professionalism in pharmacy extends beyond knowledge. Instead, students learned that patient engagement, empathy, and trust-building are fundamental to their identity as healthcare providers. Initially, many students found it challenging to balance clinical accuracy with building rapport with patients, often focusing on technical accuracy. However, through continued practice and feedback, they recognised that effective pharmacists must personalise their approach to each patient’s needs.

*“I know that each case is unique and individual, and I need to be prepared to approach a specific situation differently. This requires both confidence and experience.”*

Demonstrator and peer feedback played a significant role in shaping students’ professional identity, highlighting the importance of active listening, patient education, and adapting communication styles. As they refined these skills, students developed a stronger sense of responsibility toward patient well-being, reinforcing their commitment to ethical and patient-centred care.

*“I believe that it is important to hear out patient’s concerns and to provide empathy and reassurance towards them. I find that it is very easy for anybody to just read whatever is printed on the medication label.”*

By the end of their counselling experiences, students had a more comprehensive view of their professional identity, as not just dispensing medications, but also as accessible, empathetic healthcare professionals who play a crucial role in improving patient health outcomes.

#### 3.4.2. Opportunities to Learn About Struggles in Real-Life Practice

Demonstrators played a key role in bridging the gap between theoretical knowledge and the practical challenges of pharmacy practice. Many students found their insights invaluable in preparing them for the realities of counselling patients in busy and unpredictable environments. Students were also eager to learn from the expertise from demonstrators, as they engage with demonstrators to learn how to react in difficult real-life pharmacy situations. By learning from experienced professionals, students gained a more realistic perspective of what to expect in their future practice and how to navigate these challenges effectively.

*“I also had the chance to ask the demonstrators how they would articulate themselves in difficult situations—for example, discussing the tolerance and addiction potential associated with opioids, and the increased risk of suicidal ideation associated with initial use of antidepressants.”*

#### 3.4.3. Incorporating Feedback to Working Opportunities

Students found demonstrator feedback to be the most actionable in preparing for real-life practice, as it was structured, specific, and directly applicable to patient interactions. Demonstrators provided strategies for improving communication, ensuring information was concise yet comprehensive. Peer feedback helped students recognise how their explanations were perceived by others. Self-reflection further reinforced this learning by allowing students to assess their progress over time, making connections between their feedback and improvements in real-world pharmacy interactions. Students have reflected on their counselling approaches in real-life opportunities, such as working in community pharmacies and pharmacy placements. Many noted that applying feedback helped them navigate workplace situations with greater ease, improving their ability to communicate with patients and handle unexpected challenges. Additionally, students noted that due to the improved flow and structure from feedback and practice, they are able to adapt their counselling to fast-paced community pharmacy settings.

*“I carry the lessons I learned from my demonstrators every shift at work and even passed them onto my peers. It is essential to know the proper practice of a pharmacist as we (as health care professionals) have a duty of educating and providing care to the community.”*

#### 3.4.4. Reinforcing Skills to Self-Reflect in Future Pharmacy Practice

Students recognised that self-reflection is an essential tool for continuous improvement in their pharmacy careers. Through watching recorded videos and receiving feedback from multiple sources, self-reflection enabled them to recognise patterns in their communication, identify recurring mistakes, and implement changes in future interactions. By developing this habit, students acknowledged that practicing self-reflection prepared them for lifelong learning, ensuring they could adapt to evolving professional demands and consistently enhance their patient care approach. Moreover, students appreciated the structure of the self-reflection prompt “keep, stop, start” to highlight strengths and weaknesses.

*“Although I didn’t see it at the start, I have come to realise that self-reflection was an integral part of my learning. It was watching myself back on video that I better understood the feedback I was receiving and allowed myself to be more comfortable counselling patients as I was able to see how I came across from a third-person perspective and what to improve.”*

## 4. Discussion

To our knowledge, this is one of only a few studies to examine the combination of educator and peer feedback and self-reflection on simulated counselling activities in pharmacy students. We achieved this through a qualitative evaluation of third year pharmacy students’ reflections on their learning experiences. Our results emphasize the many ways that peer and educator feedback can create supportive learning environments and contribute to the development of patient counselling skills, and the impacts of these learning activities on students’ emerging professional identity as a pharmacist. Our findings add to the current literature on feedback, reflection, and professional identity formation in a number of ways.

Firstly, peer and demonstrator feedback were integral to students’ development of counselling skills. Many students reported that working with peers created a low-pressure learning environment, allowing them to practice freely and experiment with different counselling techniques. This aligns with previous research indicating that peer learning reduces anxiety and fosters skill development through mutual support and shared experiences [16]. However, students had differing views on the usefulness of peer feedback. While some noted that peer feedback was reassuring, others emphasised that it often lacked the depth needed for substantial improvement, suggesting that peer feedback is sometimes overly positive and lacks critical evaluation. Literature investigating the effects of these feedback mechanisms emphasise that the incorporation of self and peer assessment has been noted to enhance critical thinking and learning outcomes, but outline a number of influences on the effectiveness of peer feedback provided. These include motivational factors like satisfaction and self-efficacy [17], the influence of students’ demographic backgrounds, academic experiences, and psychological traits on shaping engagement and learning outcomes [18], as well as logistical factors, such as the timing of assignments [19]. One mechanism that has been suggested to address the variability in peer feedback is a model whereby students provide feedback on feedback received from their peers [20], such as a rating system or feedback justification to encourage accountability. Clear expectations regarding feedback and refining peer feedback mechanisms could also be useful. However, logistical barriers such as time and resource constraints can impact the implementation of these initiatives.

In contrast, demonstrator feedback was perceived as more structured and actionable, providing students with expert insights on how to improve patient engagement and communication clarity. Students valued demonstrators’ ability to provide individualised feedback, reinforcing the importance of tailored feedback in professional skill development. This is consistent with broader research on tailored feedback, which highlights its role in improving student engagement and performance [21].

Self-reflection was identified as a crucial tool in tracking progress and setting learning goals. Students found structured reflection frameworks beneficial in identifying strengths, weaknesses and areas for improvement, which aligns with Schön’s theory of reflective practice, a widely accepted approach in professional education [22]. Video review, in particular, helped students evaluate their performance objectively, allowing them to refine their counselling techniques through self-directed learning; this is consistent with previous studies such as Sargeant et al. [23]. However, some students felt that self-reflection became repetitive and less insightful over time, suggesting that its value may diminish as students become more competent. This emulates the concept of diminishing returns in learning, how initial learning phases yield significant gains, which taper off as proficiency increases [24]. Although the intrinsic motivation to self-reflect might diminish, ensuring that the value of self-reflection is reinforced in students is essential, which may address this issue to some extent. Educational activities on the importance of self-reflection, such as educational workshops and group discussions, can reinforce this and encourage engagement [25]. Additionally, some students found that informal self-reflection and immediate peer discussions during practice were more effective than formal written reflections, highlighting the importance of flexibility in reflective learning approaches [26]. The use of a range of written, reflective and dialogic feedback strategies has been shown to create a more engaging and supportive learning environment, moving away from traditional feedback practices [27].

Beyond skill acquisition, the study revealed that engaging in counselling practice and receiving feedback helped shape students’ professional identities in a variety of ways. Initially, many students viewed their role as primarily dispensing medication and providing technical information. However, through counselling simulations and feedback, they gained a deeper appreciation of the pharmacist’s role in patient care, communication and education. This finding supports previous literature emphasising the importance of experiential learning in fostering professional identity [2]. Furthermore, educators were instrumental in building confidence and providing strategies to practice addressing challenges in real life practice, highlighting the role of mentorship in professional identity formation [28]. By applying feedback in real-life settings, such as workplace placements, students developed a more patient-centred approach to counselling, which aligns with contemporary pharmacy education models advocating for active, experiential learning [29].

### Limitations

Transferability of results is a potential issue for all qualitative research, and our study is no different. A key consideration when interpreting these findings is their transferability to students enrolled in other health programs. This is especially relevant in disciplines where communication training is more extensively integrated throughout the degree, or where university-based learning is more closely connected with workplace or clinical training experiences. Barriers such as faculty workload, training requirements for effective feedback delivery, class size considerations or logistical constraints related to the structure of counselling assessments and grading may limit the ability of other institutions to implement these findings. Additionally, our results may not represent the “true” beliefs and values of our students. The reflections analysed here were completed as part of students’ assessment and, even if they were not being overtly deceptive, social desirability bias [30] may have led them to state what they thought the marker wanted to hear, rather than what they truly believed and valued. The variability of feedback quality (from both educators and peers) noted by our participants may also have influenced students’ experiences in the unit and the development of their reflective statements. Meanwhile, the use of submitted material to elicit student views meant that there was inevitably a loss of nuance, which is possible in verbal communication, and that there was no opportunity for the researchers to probe for more detail or to clarify issues if necessary. However, we discovered a rich range of opinion about the learning activities in this unit and thematic saturation was reached (with all themes complete and well-described), suggesting that these are not major issues. Finally, we focussed only the views of students. While this, in itself, does not diminish our findings, educational initiatives are best evaluated through a range of lenses [31]—including the student, teacher, and other educators—and incorporating other views could lead to a richer evaluation here.

## 5. Conclusions

This study provides new insights into the impacts of self-reflection and peer and educator feedback on the development of pharmacy students’ patient counselling skills. Our findings highlight the importance of balancing challenge and support for effective skill development and refinement, and the value of feedback and ongoing self-reflection to professional identity formation. Students perceived feedback as variable and often valued educator feedback as it was seen as authoritative, specific, and actionable. While peer feedback was perceived to be beneficial for creating a supportive learning environment, mechanisms are needed to ensure it provides information that students view as useful for their learning. Overall, integrating consistent, high-quality feedback, peer assessment, and self-reflection in pharmacy education is perceived as valuable in enhancing students’ learning experiences and preparing them for professional practice, and consideration should be given to how these strategies can be implemented in pharmacy curricula.

## Figures and Tables

**Table 1:** Reflection questions completed by students.

Timepoint	Reflection Questions
After each counselling session	What did you do well in this interaction? What can you improve? What have you learnt from this class?
End of semester reflective statement	Describe how you felt about watching yourself on video. Compare your performance from the start of semester to the end. What areas needed improvement? What helped you to improve and can you see the feedback given to you during the semester was implemented? Describe how the lessons you have learned will influence your practice. Do you believe that each of the following is a beneficial way of learning: Practice with peers, feedback from demonstrators, and self-reflection. Why or why not?

**Table 2:** Overview of identified themes and subthemes.

Theme	Subthemes
Impact of peer feedback on development of skills and confidence	Supportive peer dynamics Developing a personal counselling style through peer practice
Impact of self-reflection and assessment on development of skills and confidence	Goal setting through self-reflection Video review as a tool for skill refinement
Impact of demonstrator feedback on development of skills and confidence	Feedback variation in learning growth Addressing self-doubt in counselling certain medications
Professional identity	Learning about the value pharmacists can bring Opportunities to learn about struggles in real-life practice Incorporating feedback to working opportunities Reinforcing skills to self-reflect in future practice

## Data Availability

The original contributions presented in this study are included in the article/Supplementary Materials. Further inquiries can be directed to the corresponding author.

## Supplementary Materials

The following supporting information can be downloaded at: https://www.mdpi.com/article/10.3390/pharmacy14020041/s1, Table S1: Completed COREQ checklist.
