# Supplementary material for: Pharmacy Students’ Perceptions of Self-Reflection and Peer and Educator Feedback on the Development of Patient Counselling Skills: A Qualitative Analysis
Source: Pharmacy (Basel). 2026 Mar 3;14(2):41. doi: 10.3390/pharmacy14020041 (PMC13010727; doi:10.3390/pharmacy14020041)
Supplement: Supplementary file 1 [file pharmacy-14-00041-s001.zip › pharmacy-4158248-supplementary.pdf]

Consolidated criteria for reporting qualitative studies (COREQ): 32-item checklist

No. Item	Guide questions/description	Reported on Page #
**Domain 1: Research team and reflexivity**		
Personal Characteristics		
1. Interviewer/ facilitator	Which author/s conducted the interview or focus group?	N/A
2. Credentials	What were the researcher's credentials? E.g. PhD, MD	Initial data coding and analysis were conducted by TI, a final year female pharmacy student who had completed PHAR3825 in 2024 (page 5)
3. Occupation	What was their occupation at the time of the study?	a final year female pharmacy student who had completed PHAR3825 in 2024 (page 5)
4. Gender	Was the researcher male or female?	Female (page 5)
5. Experience and training	What experience or training did the researcher have?	TI received training in qualitative methods and thematic analysis (page 5)
Relationship with participants		
6. Relationship established	Was a relationship established prior to study commencement?	XX had no prior relationship with the participants (page 5)
7. Participant knowledge of the interviewer	What did the participants know about the researcher? e.g. personal goals, reasons for doing the research	N/A
8. Interviewer characteristics	What characteristics were reported about the inter viewer/facilitator? e.g. Bias, assumptions, reasons and interests in the research topic	N/A

**Domain 2: study design**		
Theoretical framework		
9. Methodological orientation and Theory	What methodological orientation was stated to underpin the study? e.g. grounded theory, discourse analysis, ethnography, phenomenology, content analysis	A phenomenological approach [15] underpinned the data analysis (page 5)
Participant selection		
10. Sampling	How were participants selected? e.g. purposive, convenience, consecutive, snowball	Purposive sampling (page 4)
11. Method of approach	How were participants approached? e.g. face-to-face, telephone, mail, email	Via online learning management system (page 4)
12. Sample size	How many participants were in the study?	203 student reflections (page 5)
13. Non-participation	How many people refused to participate or dropped out? Reasons?	One student did not give permission for their reflective statement to be analysed, and one did not complete the assessment, leaving a total of 201 reflective statements (99% of the cohort) for analysis (page 5).
Setting		
14. Setting of data collection	Where was the data collected? e.g. home, clinic, workplace	N/A
15. Presence of non-participants	Was anyone else present besides the participants and researchers?	N/A
16. Description of sample	What are the important characteristics of the sample? e.g. demographic data, date	203 students completed PHAR3825 in 2022. One student did not give permission for their reflective statement to be analysed, and one did not complete the assessment, leaving a total of 201 reflective statements (99% of the

		cohort) for analysis. 131 students (=65%) were female and the median age of students was 20 years. (page 5)
Data collection		
17. Interview guide	Were questions, prompts, guides provided by the authors? Was it pilot tested?	N/A
18. Repeat interviews	Were repeat inter views carried out? If yes, how many?	N/A
19. Audio/visual recording	Did the research use audio or visual recording to collect the data?	N/A
20. Field notes	Were field notes made during and/or after the interview or focus group?	N/A
21. Duration	What was the duration of the interviews or focus group?	N/A
22. Data saturation	Was data saturation discussed?	Data analysis continued until all reflective statements were analysed and thematic saturation (the point at which no new themes are emerging, and all themes are complete and well-described) [14] was reached. (page 5)
23. Transcripts returned	Were transcripts returned to participants for comment and/or correction?	N/A
**Domain 3: analysis and findings**		
Data analysis		
24. Number of data coders	How many data coders coded the data?	Initial data coding and analysis were conducted by TI, with discussion amongst the authors was used to refine codes during this interpretive process. Ongoing discussion amongst JP, AB, JLP,

		and TI was used to refine the codes and develop, define and name themes. (page 5)
25. Description of the coding tree	Did authors provide a description of the coding tree?	There is no description of the coding tree.
26. Derivation of themes	Were themes identified in advance or derived from the data?	Derived from the data (page 4)
27. Software	What software, if applicable, was used to manage the data?	QSR International Nvivo software version 12 (page 4)
28. Participant checking	Did participants provide feedback on the findings?	Participants were not invited to provide feedback on the results (page 5)
Reporting		
29. Quotations presented	Were participant quotations presented to illustrate the themes/findings? Was each quotation identified? e.g. participant number	Yes—specific comments are supported by direct quotes anonymised using participant numbers were used to identify each quote (pages 6-11)
30. Data and findings consistent	Was there consistency between the data presented and the findings?	Yes
31. Clarity of major themes	Were major themes clearly presented in the findings?	Yes
32. Clarity of minor themes	Is there a description of diverse cases or discussion of minor themes?	Yes, discussion of diverse viewpoints is provided for all categories identified where relevant
